# SET Overexpression is Associated with Worse Recurrence-Free Survival in Patients with Primary Breast Cancer Receiving Adjuvant Tamoxifen Treatment

**DOI:** 10.3390/jcm7090245

**Published:** 2018-08-28

**Authors:** Yu-Hsiang Huang, Pei-Yi Chu, Ji-Lin Chen, Chun-Teng Huang, Chia-Han Lee, Ka-Yi Lau, Wan-Lun Wang, Yu-Ling Wang, Pei-Ju Lien, Ling-Ming Tseng, Chun-Yu Liu

**Affiliations:** 1 School of Medicine, National Yang-Ming University, Taipei 11221, Taiwan; yuhsiang83@gmail.com (Y.-H.H.); DAK17@tpech.gov.tw (C.-T.H.); 2 Department of Pathology, Show Chwan Memorial Hospital, Changhua City 50091, Taiwan; chu.peiyi@msa.hinet.net; 3 School of Medicine, College of Medicine, Fu Jen Catholic University, New Taipei City 24205, Taiwan; 4 Comprehensive Breast Health Center, Taipei Veterans General Hospital, Taipei 11217, Taiwan; jlchen_@outlook.com (J.-L.C.); rabbitwang78tw@gmail.com (W.-L.W.); away390@yahoo.com.tw (Y.-L.W.); prlain@vghtpe.gov.tw (P.-J.L.); 5 Division of Medical Oncology, Department of Oncology, Taipei Veterans General Hospital, Taipei 11217, Taiwan; gahanleeo@gmail.com (C.-H.L.); cayeelau@gmail.com (K.-Y.L.); 6 Division of Hematology & Oncology, Department of Medicine, Yang-Ming Branch of Taipei City Hospital, Taipei 11221, Taiwan; 7 Division of Experimental Surgery, Department of Surgery, Taipei Veterans General Hospital, Taipei 11217, Taiwan; 8 Department of Nursing, Taipei Veterans General Hospital, Taipei 11217, Taiwan; 9 Division of Transfusion Medicine, Department of Medicine, Taipei Veterans General Hospital, Taipei 11217, Taiwan

**Keywords:** breast cancer, tamoxifen, SET, CIP2A, PP2A

## Abstract

Adjuvant tamoxifen reduces the recurrence rate of estrogen receptor (ER)-positive breast cancer. Previous in vitro studies have suggested that tamoxifen can affect the cancerous inhibitor of protein phosphatase 2A (CIP2A)/protein phosphatase 2A (PP2A)/phosphorylation Akt (pAkt) signaling in ER-negative breast cancer cells. In addition to CIP2A, SET nuclear proto-oncogene (SET) oncoprotein is another intrinsic inhibitor of PP2A, participating in cancer progression. In the current study, we explored the clinical significance of SET, CIP2A, PP2A, and Akt in patients with ER-positive breast cancer receiving adjuvant tamoxifen. A total of 218 primary breast cancer patients receiving adjuvant tamoxifen with a median follow-up of 106 months were analyzed, of which 17 (7.8%) experienced recurrence or metastasis. In an immunohistochemical (IHC) stain, SET overexpression was independently associated with worse recurrence-free survival (RFS) (hazard ratio = 3.72, 95% confidence interval 1.26–10.94, *p* = 0.017). In silico analysis revealed mRNA expressions of *SET*, *PPP2CA*, and *AKT1* significantly correlated with worse RFS. In vitro, SET overexpression reduced tamoxifen-induced antitumor effects and drove luciferase activity in an Estrogen receptor element (ERE)-dependent manner. In conclusion, SET is a prognostic biomarker in patients with primary ER-positive breast cancer receiving adjuvant tamoxifen and may contribute to the failure of the tamoxifen treatment by modulating the ER signaling. Our study warrants further investigation into the potential role of SET in ER-positive breast cancer.

## 1. Introduction

Estrogen receptor (ER)-positive breast cancer accounts for 70% of breast cancer subtypes and, despite its relatively good prognostic tumor biology, harbors a substantial and constant risk of recurrence after the primary resection, even after five years of treatment [1,2,3]. Adjuvant hormonal therapies, such as tamoxifen and aromatase inhibitors, have significantly reduced the risk of recurrence in patients with ER-positive early breast cancers [4,5]. Several randomized controlled trials of hormonal therapy have demonstrated that five years of adjuvant tamoxifen safely improves 15-year recurrence-free survival (RFS) and overall survival in breast cancer patients [6,7,8,9]. However, patients receiving adjuvant tamoxifen would still experience breast cancer recurrence; according to the meta-analysis by the Early Breast Cancer Trialists' Collaborative Group, five years of adjuvant tamoxifen safely reduces 15-year risks of breast cancer recurrence and death, with the cumulative recurrence rate reaching 33% in 10,645 women (100% ER-positive, 44% node-positive, 51% chemotherapy) [6]. Tamoxifen acts as a selective estrogen receptor modulator and, despite its relatively inferior efficacy to aromatase inhibitors in terms of reducing risk of recurrence in post-menopausal ER-positive breast cancers, continues to demonstrate a major role when it is used for an extended period in the adjuvant setting [10,11]. The impressive Adjuvant Tamoxifen: Longer Against Shorter (ATLAS) and the adjuvant Tamoxifen—To offer more (aTTom) trials both indicated that 10 years of tamoxifen is superior to five years of other treatments [10,11]. There have been studies exploring factors associated with the recurrence of ER-positive early breast cancers, including more recent molecular studies [2,6,12,13,14]. Sestak et al. demonstrated that the traditional four immunohistochemical markers (IHC4 score) could not provide prognostic information in years 5–10, except for nodal status and tumor size, and the molecular-based risk of recurrences (ROR) gene panel gave the strongest prognostic information in years 5–10 [13]. The Oncotype DX 21-gene assay quantifies the risk of distant recurrence in women with ER-positive, lymph node-negative breast cancer treated with adjuvant tamoxifen and has been validated to predict the benefit from chemotherapy in this population [15,16]. Collectively, the usefulness of these multi-gene testing techniques suggests that molecular markers may help to identify patients at risk and select patients who could benefit most from hormonal therapy. 

Protein phosphatase 2A (PP2A), a cellular serine/threonine protein phosphatase, can act as a tumor suppressor by direct dephosphorylation of oncogenic kinases, such as Akt, c-Myc, Bcl-2, β-catenin, MAPK, and ERK [17,18,19,20]. In our previous study, we found that tamoxifen induced apoptosis by downregulating the cancerous inhibitor of protein phosphatase 2A (CIP2A), increasing PP2A activity, and suppressing phosphorylation Akt (pAkt) signaling in ER-negative breast cancer cells [21]. CIP2A is an oncogenic cellular inhibitor of PP2A, and overexpression of CIP2A inhibits PP2A, thereby causing various kinase-driven signaling activations, which promote tumor growth and aggressiveness [22,23,24]. All this suggests that the inhibition of the PP2A tumor suppressor activity may signal a poor clinical outcome for the tamoxifen treatment.

The SET protein, also known as I2PP2A or template activating factor-1 (TAF-1), is another oncogenic cellular inhibitor of PP2A by direct binding to PP2AC (the catalytic subunit) and plays a role in modulating tumor progression and metastasis [25,26,27,28]. The overexpression of SET contributes to the tumor cell proliferation, metastasis, and drug resistance [29]; on the other hand, the knockdown of SET or the administration of the SET antagonist fingolimod (FTY720), thereby restoring PP2A antitumor activity, reduces the clonogenic and tumorigenic potential in vitro and in vivo [19,30,31].

In this study, we aimed to investigate the associations of four biomarkers, including SET, CIP2A, PP2A, and Akt, with recurrence-free survival in ER-positive breast cancer patients receiving adjuvant tamoxifen therapy.

## 2. Experimental Section

### 2.1. Patients

This study was approved by the ethics committee of the Institutional Review Board of Taipei Veterans General Hospital (Protocol No. 2013-10-005A; date of approval: 4 November 2013 and Protocol No. 2017-10-016AC; date of approval: 28 October 2017) and was conducted in compliance with the Helsinki Declaration. The patients were enrolled between 13 November 2000 and 10 December 2010. The last follow-up date was 24 February 2016. Primary breast tumor specimens were obtained from 218 patients diagnosed with ER-positive breast cancer, who underwent tamoxifen therapy after surgery. The exclusion criteria included a previous history of neoadjuvant antiestrogen therapy, a primary non-operable tumor, and metastasis before tamoxifen treatment. All the patients underwent tamoxifen treatment with a median follow-up of 106 months. The tumor-node-metastasis (TNM) staging, histological grade, and tumor type were determined in accordance with the World Health Organization (WHO) classification system. The clinical data were collected from medical records by oncologists. Representative areas of each tumor were carefully selected and constructed into tissue microarrays (TMA). The TMA from the tumor samples of patients with ER-positive primary breast cancers were prepared as previously described [32]. 

### 2.2. Immunohistochemistry

Immunohistochemical analysis of the biomarkers on 4-µm paraffin-embedded breast cancer tissue sections were performed in accordance with a standard protocol using commercially available antibodies. The TMA slides were deparaffinized and rinsed with 10 mM of Tris-HCl (pH 7.4) and 150 mM of sodium chloride. Peroxidase was quenched with methanol and 3% hydrogen peroxide. The slides were placed in 10 mM of citrate buffer (pH 6.0) at 100 °C for 20 min in a pressurized heating chamber. Afterwards, the slides were incubated with the following antibodies for 1 h at room temperature, including the SET antibody with a dilution of 1:500 (A302-262A, Bethyl Laboratories, Montgomery, TX, USA), the CIP2A antibody with a dilution of 1:100 (ab84547; Abcam, Cambridge, MA, USA), the pAkt antibody with a dilution of 1:200 (pAkt1/2/3 Thr308, sc-16646-R; Santa Cruz Biotechnology, Dallas, TX, USA), and the pPP2A antibody with a dilution of 1:100 (ab32104, Abcam). The bound antibodies were detected using the EnVision Detection Systems Peroxidase/DAB, Rabbit/Mouse kit (Agilent, Santa Clara, CA, USA). The slides were counterstained with hematoxylin. The negative controls had the primary antibodies replaced by phosphor-buffered saline. A mouse monoclonal antibody was used to detect ER (Dako), and the tissues were identified as ER-positive breast cancer if more than 10% of the nuclei were stained.

### 2.3. Determination of Histology Score (H-Score)

The IHC staining of each biomarker was assessed semi-quantitatively by assigning an H-score (ranging 0–300), which was defined by multiplying the percentage of positive-stained carcinoma cells (from 0–100) by the staining intensity (from negative staining as 0, weak as 1, moderate as 2, to strong staining as 3), as described previously [24]. A pathologist specializing in breast cancer pathology performed the assessment of the H-scores independently without being given the clinical information on the tumor specimens. A receiver operating characteristic (ROC) curve analysis was performed for the selection of cutoffs of each biomarker H-score for defining low versus high expression tumors and clinical-pathological correlation as described in Section 2.10. 

### 2.4. In Silico Survival Analysis with Public Open Database

An analysis of the Kaplan–Meier survival curve between gene expression and RFS was evaluated from a public available platform (http://kmplot.com) [33]. A group of patients (*n* = 1043) were selected with the inclusion criteria including ER-positive and tamoxifen-treated patients only. The RFS curves with the auto-selected best cutoff were plotted based on the gene expression of *SET* (Afftymetrix probe ID 200631), *KIAA1524* (ID 231855), *PPP2CA* (ID 208652), and *AKT1* (ID 207163).

### 2.5. Reagents and Antibodies

The tamoxifen used for the in vitro experiments was purchased from Cayman Chemical (Ann Arbor; MI, USA). The tamoxifen was dissolved in dimethyl sulfoxide (DMSO) at different concentrations, and the final DMSO concentration was 0.1% after being added to the medium. Dulbecco’s modified Eagle’s medium (DMEM) and fetal bovine serum were obtained from GIBCO (Invitrogen, Carlsbad, CA, USA). For immunoblotting, the anti-beta Actin antibody (Cat No. ab6276) was purchased from Abcam (Cambridge, MA, USA). The antibody against poly (ADP-ribose) polymerase (PARP; Cat No. 9532) and DYKDDDDK Tag (Cat No. 2368) were purchased from Cell Signaling Technology (Danvers, MA, USA).

### 2.6. Cell Culture and Western Blot Analysis

The human MCF7 (ATCC HTB-22) luminal breast carcinoma cell line was obtained from American Type Culture Collection (Manassas, VA, USA) and routinely grown in DMEM supplemented with 10% fetal bovine serum, 0.1 mM of nonessential amino acids, 2 mM of L-glutamine, 100 U/mL of penicillin G, 100 µg/mL of streptomycin sulfate, and 25 µg/mL of am*p*hotericin B in a 37°C humidified incubator containing a 5% CO_2_ atmosphere. The lysates of breast cancer cells for immunoblotting were treated with drugs at the indicated concentrations for various periods of time. Cell apoptosis was assessed by PARP cleavage of western a blot. Western blot analysis was performed as previously reported [21].

### 2.7. Transient Transfection 

The MCF7 breast cancer cells were plated in 1 × 10^6^ cells/dish in a 6-cm culture dish. The Myc-DDK-tagged-human SET expression plasmid was kindly provided by Kuen-Feng Chen [34]. Aliquots containing 4 µg of the SET expression or the control pCMV6 GFP plasmids (PS100010; OriGene, Rockville, MD, USA) in 500 μL of Opti-MEM were used for lipid-mediated transfection with Lipofectamine3000 (Invitrogen, Waltham, MA, USA) according to the manufacturer’s instructions. On the next day, the cells were treated at the indicated drug concentrations for a period of time and both floating and adherent cells were harvested for immunoblotting. 

### 2.8. Cell Viability Assay

The cells were plated in a 96-well plate in 150 μL of DMEM per well and cultured for 24 h. The MCF7 breast cancer cells were treated with or without tamoxifen at the indicated drug concentration for 72 h. The cell viability was assessed by colorimetric assay using 3-(4,5-dimethylthiazol-2-yl)-2,5-diphenyltetrazolium bromide (MTT). The cells were incubated for 3 h at 37 °C with MTT; after incubation, the medium was removed, and cells were treated with DMSO for 5 min. The viability was evaluated by ultraviolet absorption spectrum at 570 nm with a Bio-Tek uQuant microplate spectrophotometer (BioTek Instruments, Winooski, VT, USA).

### 2.9. Dual Luciferase Assay

The MCF7 cells were transfected by Lipofectamine3000 (Invitrogen, Thermofisher) with 3X estrogen-response element (ERE) TATA luc, firefly luciferase reporter construct, and reference pCMV-renilla along with either the SET expression plasmid or the control pCMV6 GFP plasmid. The 3X ERE TATA luc was a gift from Donald McDonnell (plasmid # 11354; Addgene, Cambridge, MA, USA) [35]. After 24 h, the cells were treated with and without 1 nM of estradiol (E2) and maintained for an additional 24 h. The promoter activity was analyzed by dual luciferase assay, according to the manual description (Promega, Madison, WI, USA).

### 2.10. Statistical Analysis

The statistical analysis was performed with SPSS 24.0 software (SPSS, Chicago, IL, USA). RFS was defined as the time from surgery until any primary or distant recurrence with an appearance of a secondary tumor or death. The ROC curve analysis was used to calculate the optimal cutoff value of the H-scores for the aforementioned biomarkers SET, CIP2A, pPP2A, and pAkt. Using the ROC curve determined cutoff value, two risk subgroups categorized as low and high were established for each individual biomarker. The survival curves were plotted using the Kaplan–Meier method and compared with the log-rank test. The Cox regression was applied for multivariate analysis taking into consideration the relevant significant parameters in the univariate analysis. A *p*-value of less than 0.05 was considered statistically significant.

## 3. Results

### 3.1. Clinical Significance of Biomarkers SET and CIP2A in Patients with Breast Cancer

**Figure 1:**
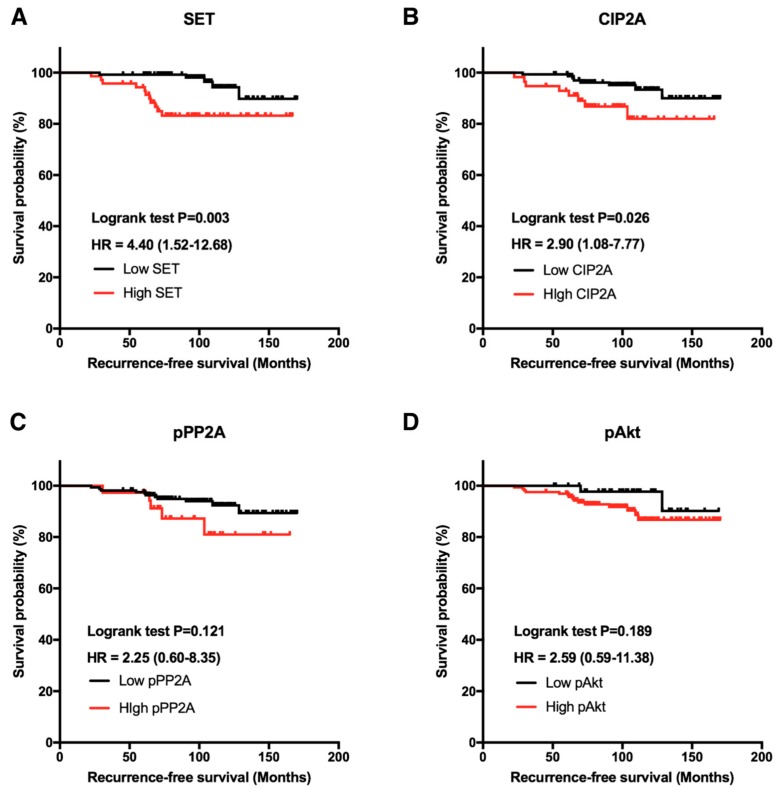
Clinical significance of protein biomarkers in human breast cancer patients. Kaplan–Meier analysis of the influence of SET (**A**), CIP2A (**B**), pPP2A (**C**), and pAkt (**D**) expression on recurrence-free survival (RFS) in patients with estrogen receptor (ER)-positive breast cancer receiving adjuvant tamoxifen.

We examined 218 ER-positive specimens collected from primary breast cancer patients with a median age of 50.6 years (interquartile range: 43–58 years; Table 1). These patients were later treated with adjuvant tamoxifen as hormonal therapy for a medium duration of 57.6 months (interquartile range: 31.3–67.8 months), and we observed the clinical outcomes until death, censorship, or loss to follow-up. With a median follow-up of 106 months, 17 (7.8%) patients suffered from a recurrence or metastasis. Based on the ROC curve analysis, the cutoff values of the H-scores of SET, CIP2A, pPP2A, and pAkt were selected as 130, 195, 205, and 95, respectively. The correlation between RFS and the biomarkers revealed that those with high SET expression had poorer RFS than those with low SET expression (*p* = 0.003; Figure 1A). Similarly, high CIP2A expression is associated with worse RFS (*p* = 0.026; Figure 1B). In contrast, pPP2A and pAkt showed only trends towards worse RFS (*p* > 0.05; Figure 1C,D). 

### 3.2. Biomarker SET is an Independent Prognostic Predictor in ER-Positive Breast Cancer Receiving Adjuvant Tamoxifen Treatment

**Figure 2:**
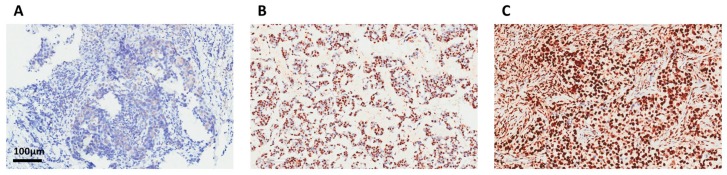
Immunohistochemical stain for SET. Representative images for 1+ expression in breast cancer specimen (**A**); 2+ expression in breast cancer specimen (**B**); 3+ expression in breast cancer specimen (**C**).

Representative immunohistochemical stainings of SET in the breast cancer tissue are illustrated (Figure 2). Taking into consideration the clinico-pathological characteristics in the multivariate analysis by Cox regression, high SET expression was independently associated with poor RFS (*p* = 0.017, hazard ratio (HR) = 3.72, CI = 1.26–10.94; Table 2). We further divided patients into high- and low-expression SET subgroups (32.6% vs. 67.4%) based on a cutoff value of 130, as previously described, and examined whether there was unequal distribution of clinicopathological factors between the two groups. Accordingly, no statistically significant correlations of age, grade, stage, or presence of lymphovascular invasion with SET expressions were found (Table 3). Although CIP2A demonstrated a statistical significance in predicting RFS in the univariate analysis (*p* = 0.026), the *p*-value was not significant in the multivariate analysis (*p* = 0.173). These results demonstrated that SET is an independent prognostic predictor in ER-positive primary breast cancer receiving adjuvant tamoxifen treatment.

### 3.3. In Silico Analysis of Correlation of SET Gene Expression with Outcome in Public Data

**Figure 3:**
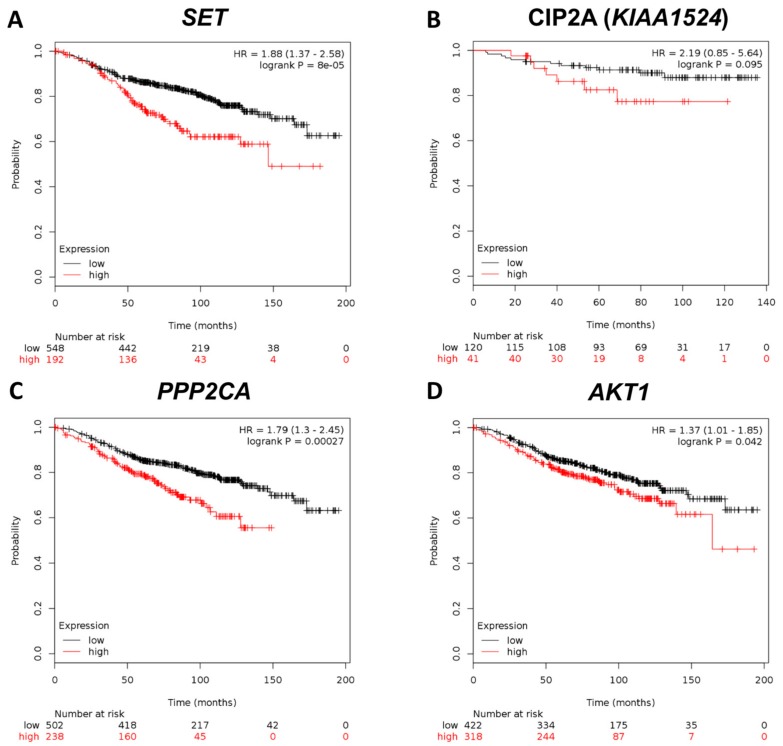
Clinical significance of gene biomarkers in human breast cancer patients. Kaplan–Meier analysis of the influence of SET (**A**), KIAA1524 (**B**), PPP2CA (**C**), and AKT1 (**D**) expression on RFS in patients with ER-positive breast cancer receiving adjuvant tamoxifen from the public platform (http://kmplot.com).

To further decipher the clinical relevance of these biomarkers, we searched the publicly available database Kaplan–Meier Plotter (http://kmplot.com), which explores the biomarkers in gene expression using large samples of breast tumor patients (*n* = 3951) [33]. Patients with ER-positive breast cancer receiving tamoxifen-only treatment (*n* = 1043) were selected, and RFS survival analysis was performed in the gene expression with the auto-selected best cutoff. The results disclosed that breast cancer patients with *SET* gene overexpression had worse RFS compared with those with low *SET* gene expression (*p* < 0.001, HR = 1.88, CI = 1.37–2.58; Figure 3A). Nonetheless, the *KIAA1524* gene, which encodes protein CIP2A, showed no statistical significance in predicting the clinical outcome (*p* = 0.095; Figure 3B). *PPP2AC* and *AKT1* demonstrated poor prognosis in ER-positive breast cancer patients (*p* <0.001 and *p* < 0.05; Figure 3C,D). Overall, the public database results supported our findings that *SET* overexpression correlated with worse RFS in ER-positive breast cancer patients treated with tamoxifen.

### 3.4. Overexpression of SET Suppressed the Anti-Cancer Effects of Tamoxifen on MCF7 Cells

**Figure 4:**
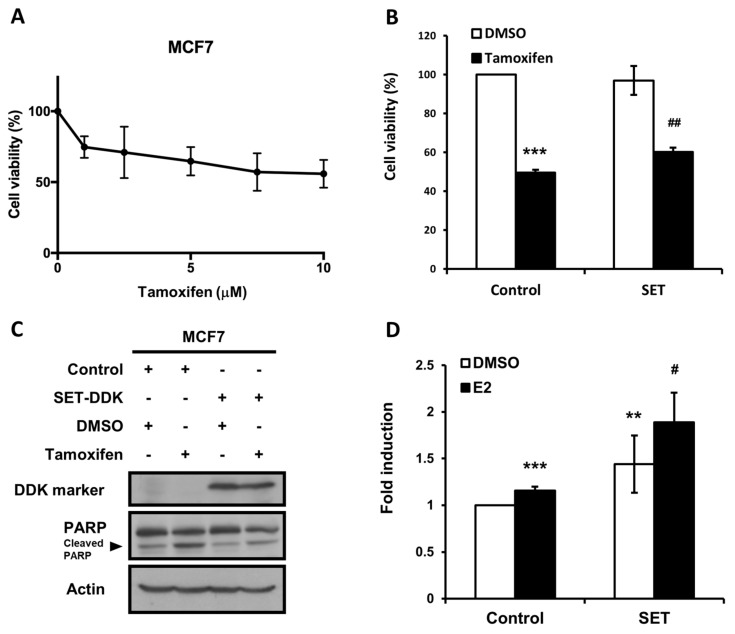
The overexpression of SET reduced tamoxifen-induced anti-proliferation and enhanced estrogen-promoted transactivation in the MCF7 cells. (**A**) 3-(4,5-dimethylthiazol-2-yl)-2,5-diphenyltetrazolium bromide (MTT) assay showing that tamoxifen exerts anti-proliferative effects in the MCF7 cells in a dose-dependent manner. (**B**) MTT assay showing that SET overexpression downregulated the anti-proliferative effects after the tamoxifen treatment. *** *p* < 0.001 compared with cells transfected with pCMV6-Entry control plasmids and treated with DMSO. ## *p* <0.01 compared with cells transfected with the SET expression plasmid and treated with DMSO. (**C**) Western blot showing that SET overexpression reduced poly (ADP-ribose) polymerase (PARP) cleavage after the tamoxifen treatment. (**D**) Estrogen receptor element (ERE)-dependent transactivation showing that SET overexpression enhanced the expression of reporter assay. ** *p* < 0.01, *** *p* < 0.001 compared with cells transfected with pCMV6-Entry (control) and treated with DMSO. # *p* < 0.05 compared with cells transfected with the SET expression plasmid and treated with estradiol (E2).

To examine the tumor suppression ability of tamoxifen on ER-positive breast cancer, we first assessed the dose-dependent antiproliferative effect of tamoxifen on ER-positive human MCF7 cells (Figure 4A). We next examined the role of SET in tamoxifen-treated breast cancer cells by ectopic overexpression of SET in the MCF7 cells followed by the tamoxifen treatment. As shown in Figure 4, the anti-proliferation induced by tamoxifen was suppressed by SET overexpression (Figure 4B). Moreover, tamoxifen-induced apoptosis, as evident by PARP cleavage, was also suppressed by SET overexpression (Figure 4C). We further examined whether SET affected the ERE-mediated effects of estrogen signaling. ERE-dependent luciferase activity bearing reporter plasmids were co-transfected with SET expression or the control plasmids into the MCF7 cells in the absence or presence of estradiol. Our results showed that the overexpression of SET in the MCF7 cells elicited the ERE-dependent luciferase activity, which was further enhanced by the presence of estradiol (Figure 4D). Taken together, the overexpression of SET suppressed tamoxifen-induced anti-cancer effects and upregulated ERE-dependent ER signaling transactivation, indicating that SET may be associated with the failure of tamoxifen treatment in ER-positive breast cancer, at least, in part, through modulating the ER signaling pathway. 

## 4. Discussion

In the present study, we found that high expression of SET or CIP2A was associated with worse RFS in patients with ER-positive primary breast cancer receiving the adjuvant tamoxifen treatment (Figure 1). In addition, SET expression was an independent prognostic factor in RFS after adjusting for clinico-pathological characteristics. Furthermore, the public database Kaplan–Meier Plotter also disclosed that higher SET gene expression, which encodes oncoprotein SET, was associated with poor RFS with primary breast cancer receiving the adjuvant tamoxifen treatment (Figure 3). The in vitro results demonstrated that ectopic-expressed SET reduced tamoxifen-induced antitumor effects. Moreover, the overexpression of SET enhanced ERE-dependent transactivation (Figure 4D). Taken together, these results suggested that SET correlates with a poor clinical outcome and may be an independent prognostic predictor in primary ER-positive breast cancer patients receiving the tamoxifen treatment.

Various studies have shown a preferential overexpression of SET in chronic myelogenous leukemia [37], head and neck squamous cell carcinoma [38], non-small cell lung cancer [31], colorectal cancer [39], and breast cancer [19]. Mahnaz et al. [19] have described that greater than 50% of breast cancer patients have SET overexpression, but there are no specific subtypes of breast cancer associated with increased SET levels. In our study, we discovered a correlation between increased SET expression and poor clinical outcomes in the subgroup of patients with primary ER-positive breast cancer receiving the adjuvant tamoxifen treatment.

The mechanisms of hormonal therapy resistance are multifaceted and vary by single or combined therapy [30]. Other than the conventional theory that tamoxifen affects cancer cell survival through estrogen receptor antagonism, our previous study showed that tamoxifen also exerts an “off-target” mechanism of apoptosis induction via the inhibition of CIP2A, an oncogenic cellular inhibitor of PP2A in ER-negative breast cancer cells [21]. Interestingly, SET is an intrinsic PP2A-interacting inhibitor [19], whereas it has been reported that PP2A could also dephosphorylate ER on S118, thereby inhibiting the ER–ERE binding activity and the ER-induced transcriptional activation of target genes [40]. Our data showed that SET overexpression enhanced ERE-mediated ER activity (Figure 4D), suggesting that SET could possibly interfere with the ER signaling pathways and contribute to tamoxifen resistance in breast cancer, at least, in part, via the inhibition of PP2A activity. However, the exact mechanisms by which SET overexpression participates in modulating ER signaling remains unknown. Although some off-target effects of increased SET on cell survival may also exist in this transient transfection system (Figure 4B,C), we further demonstrated that he overexpression of SET indeed increased ER-related activity (Figure 4D). Because tamoxifen acts as a selective ER modulator, it is also likely that increased SET, through yet-to-be determined detailed mechanisms, antagonizes tamoxifen-related ER suppression. Moreover, our study is limited by the fact that only one ER-positive MCF-7 cell line was tested for the SET–ER interacting phenomenon. Future studies using other ER-positive cell lines, including tamoxifen-resistant cells, as well as other breast cancer subtypes, such as MDA-MB-231 and MCF 10A cells as the control groups, would be helpful in deciphering the SET–ER interacting mechanisms and to confirm that our findings are specific for ER-positive breast cancers. Furthermore, the in vitro phenomenon seen in our study should be further validated by more future studies on clinical samples.

Notably, the involvement of SET in drug resistance was demonstrated in other studies. SET-induced paclitaxel resistance was found in MCF7 cells with the activation of the SET/ phosphoinositide 3-kinase (PI3K)/Akt pathway [41]. Samanta et al. showed SET expression was associated with imatinib resistance in lung cancer by the Jak2 and SET-PP2A-SHP1 pathway [42]. The elucidation of the role of the endogenous cellular inhibitor SET suggests a possible mechanism to overcome drug resistance in cancer.

## 5. Conclusions

Our results showed that SET overexpression at both the protein and gene level correlated with poor RFS in primary ER-positive breast cancer receiving tamoxifen. Moreover, SET may be associated with the failure of the tamoxifen treatment in ER-positive breast cancer, at least in part, through modulating the ER signaling pathway. Our study implicated the potential role of SET as a biomarker for selecting patients who might not experience long-term benefits from tamoxifen therapy. Our study also warrants further investigation to explore the molecular mechanisms underpinning the correlation between the ER signaling pathway and the oncoprotein SET.

## Figures and Tables

**Table 1:** General characteristics of 218 estrogen receptor (ER)-positive breast cancer patients receiving adjuvant tamoxifen treatment.

Characteristic	*n* = 218	%
Age, years (Median, IQR) ^1^	50.6 (43.0 to 58.0)	
Adjuvant Tamoxifen treatment, months (Median, IQR)	57.6 (31.3 to 67.8)	
Tumor stage (T) ^2^		
Tis	6	2.8
T1	96	44.0
T2	112	51.4
T3	3	1.4
T4	1	0.5
Nodal status (N)		
N0	158	72.5
N1	42	19.3
N2	12	5.5
N3	6	2.8
AJCC tumor-node-metastasis (TNM) Stage		
DCIS	6	2.8
I	78	35.8
II	115	52.8
III	19	8.7
Lymphovascular invasion		
Absent	189	86.3
Present	29	13.7
Histologic grade		
1	36	16.5
2	146	67.0
3	36	16.5
SET (Median, IQR) (*n* = 203) ^3^	80 (0 to 160)	
CIP2A (Median, IQR) (*n* = 202) ^3^	134 (80 to 200)	
pPP2A (Median, IQR) (*n* = 202) ^3^	133 (70 to 185)	
pAkt (Median, IQR) (*n* = 212) ^3^	142 (100 to 180)	

^1^ IQR, interquartile range; DCIS, ductal carcinoma in situ.^2^ Stage was according to 7th edition of AJCC staging [36].^3^ Data were expressed as the histology score (H-score), as described in Section 2.3.

**Table 2:** Univariate and multivariate Cox analysis of factors associated with recurrence-free survival (RFS) in ER-positive breast cancer patients receiving the tamoxifen treatment.

	Univariate RFS Analysis		Multivariate RFS Analysis	
	Hazard Ratio (95%CI)	*p*	Hazard Ratio (95%CI)	*p*
SET H-score (>130 vs. ≤130)	4.40 (1.52–12.68)	0.003	3.72 (1.26–10.94)	0.017
CIP2A H-score (>195 vs. ≤195)	2.90 (1.08–7.77)	0.026	2.08 (0.72–6.00)	0.173
Lymphovascular invasion (present vs. absent)	2.51 (0.87–7.27)	0.088	2.85 (0.97–8.38)	0.057
Grade (3 vs. 2–1)	2.05 (0.72–5.82)	0.177	2.17 (0.73–6.45)	0.162
Stage (III vs. II–I-DCIS)	2.33 (0.66–8.16)	0.184	2.15 (0.46–10.08)	0.330

**Table 3:** Association of SET expression with clinico-pathological characteristics in 203 breast cancer patients.

Characteristics	Number of Patients (*n* = 203)	SET Expression		*p*-value
		Low (*n* = 132)	High (*n* = 71)	
Age				
≤60	161	107 (81.1)	54 (76.1)	0.401
>60	42	25 (18.9)	17 (23.9)	
Tumor stage				
Tis	6	5 (3.8)	1 (1.4)	0.818
T1	92	60 (45.5)	32 (45.1)	
T2	101	64 (48.5)	37 (52.1)	
T3	3	2 (1.5)	1 (1.4)	
T4	1	1 (0.8)	0 (0)	
Nodal status				
N0	150	96 (72.7)	54 (76.1)	0.068
N1	37	21 (15,9)	16 (22.5)	
N2	11	10 (7.6)	1 (1.4)	
N3	5	5 (3.8)	0 (0)	
TNM Stage				
DCIS + I	81	56 (42.4)	25 (35.2)	0.317
II+III	122	76 (57.6)	46 (64.8)	
Grade				
1 + 2	172	117 (88.6)	55 (77.5)	0.035
3	31	15 (11.4)	16 (22.5)	
Lymphovascular invasion				
Absent	172	115 (89.1)	57 (83.8)	0.286
Present	25	14 (10.9)	11 (16.2)	
NA ^1^	6			
Tumor necrosis				
Absent	168	112 (85.5)	56 (78.9)	0.230
Present	34	19 (14.5)	15 (21.1)	
NA	1			
CIP2A				
Low (<195)	142	104 (80.6)	39 (54.9)	<0.001
High (>195)	57	25 (19.4)	32 (45.1)	
NA	3			
pPP2A				
Low (<205)	164	112 (86.2)	52 (73.2)	0.024
High (>205)	37	18 (13.8)	19 (26.8)	
NA	2			
pAkt				
Low (<195)	45	29 (22.7)	16 (22.9)	0.974
High (>195)	153	99 (77.3)	54 (77.1)	
NA	5			

^1^ NA, not available.
